# Patients refusing a permanent colostomy for rectal cancer: clinical outcome and psychological aspects

**DOI:** 10.1007/s00520-025-09837-4

**Published:** 2025-08-09

**Authors:** Nitzan Goldberg, Mika Appelbaum, Yaron Rudnicki, Hagai Soback, Assaf Rahmani, Shmuel Avital

**Affiliations:** 1 Department of Surgery, Meir Medical Center, Kfar Saba, Israel; 2 Faculty of Medicine, Tel Aviv University, Tel Aviv, Israel

**Keywords:** Colostomy, Stoma, Rectal cancer, Abdominal-perineal resection

## Abstract

**Purpose:**

For locally advanced rectal tumors affecting the lower rectum, anal sphincter, or levator muscles, abdominoperineal resection (APR) is one of the surgical options. This involves extensive tissue removal and the creation of a permanent colostomy. However, colostomy is perceived by certain patients as a serious challenge, affecting multiple aspects of their lives. Thus, some refuse surgery and accept the risks of leaving the tumor untreated. Our study aims to explore refusal rates and reasons for declining surgery.

**Methods:**

Patients who were recommended to undergo APR surgery as part of their rectal cancer treatment were evaluated. These patients were followed up and underwent interviews and completed three questionnaires: one to explore reasons for surgery refusal, another to assess depression (PHQ-9), and a third to evaluate coping strategies (COPE). A medical psychologist analyzed the questionnaire responses.

**Results:**

During the study period 96 patients were diagnosed with rectal cancer. Eighty-four patients had anterior resection, one patient had TAMIS, and 11 patients were recommended to undergo APR. Out of them, five completed the surgery, and six refused. They were the focus of our study. All patients received neoadjuvant therapy. Average follow up time was 27 months. Two patients died during the study. Primary reasons for rejecting colostomy included concerns about social stigma and feelings of shame, as well as struggles with depression and anxiety. Notably, one patient was diagnosed with moderate depression based on the PHQ-9 questionnaire.

**Conclusion:**

A planed permanent colostomy presents a significant psychological and emotional hurdle for patients. The fear of the procedure is so intense that some patients choose to avoid it while risking their lives. It is imperative to implement various measures to aid patients in the peri-operative setting, such as establishing support groups and providing mentors who have experienced the same disease and treatment.

## Introduction

Colorectal cancer is the fourth most diagnosed cancer in the USA and stands as the second leading cause of cancer related mortality [1]. Traditionally, abdominoperineal resection (APR), used to be a valid option for low-lying rectal cancer. However, APR has significantly declined in the last decades, largely due to the introduction of neoadjuvant treatment [2]. The use of total neoadjuvant treatments (TNT) have led to increased sphincter saving procedures as well as complete organ preservation in cases of complete response [3]. The evolution of local surgical techniques such as transanal endoscopic microsurgery (TEM) and transanal minimally invasive surgery (TAMIS) enabled local resection for selected patients with early-stage rectal cancer [4]. In cases of microsatellite instability high (MSI-H) tumors, an operation can be avoided completely [5]. Moreover, even in very low tumors, a sphincter preserving operation such as inter-sphincteric dissection can offer preservation of the sphincter with similar oncological outcomes [6].

Nevertheless, despite the evolving treatments, for certain patients the need for APR is inevitable—for example ultra-low rectal tumors with no sufficient response to neoadjuvant treatments, involvement of the external sphincter or invasion of the levator ani complex, and those with poor anal sphincter function [7].

A permanent colostomy could present a challenge in terms of both physiological and psychological effect, potentially impacting patients’ quality of life [8]. Various studies have demonstrated a negative psychological effect on the quality of life due to ostomy related issues such as sexual function, depression, dissatisfaction with appearance, worry about noises and more [9]. Patients are willing to accept a greater risk of local recurrence and positive margins, and substantial change in bowel movements to avoid APR surgery and permanent stoma [10].

Estimated refusal rate for rectal surgery in general (not specifically APR) is between 2.6 and 3.5% among all eligible patients diagnosed with rectal cancer [11–13]. There are several possible reasons for this refusal including avoidance from a radical surgery, concerns regarding the quality of life, personal values, and more [13–15]. Nevertheless, these investigations were carried out among individuals diagnosed with rectal cancer who were considered for various rectal surgeries, including anterior resection (AR), and not necessarily leading to a permanent colostomy. The precise rate at which patients decline APR surgery due to the planned permanent stoma is unclear, and so are the primary reasons for such refusals.

The aims of this case series were to evaluate the outcome of patients that were recommended to undergo an APR but refused and to provide additional insights regarding the psychological aspects behind their decisions.

## Methods

A retrospective and qualitative study was conducted. The study included patients that were diagnosed with rectal cancer between 2019 and 2023 and were advised for APR as part of their oncological treatment. Patients that refused the surgery were included. A long-term follow-up and interviews were conducted with these patients.

### Ethical approval

This study was performed in line with the principles of the Declaration of Helsinki. Approval was granted by the Ethics Committee of Meir Medical Center. Approval number- 0112–24-MMC.

### Patients’ recruitment and data collection

All patients were recruited from the outpatient clinic of the surgical department of our medical center. A consent form was not required to participate in the study; simply filling out the questionnaire was considered informed consent. Data was collected from the electronic charts of the patients. The information collected included background characteristics and data regarding the course of malignant disease.

### Interviews

The interviews included semi-structured interviews with the patients and three questionnaires. The interviews were conducted by a physician and by the psychologist of our department (M.A.). The semi-structured interview included informational questions regarding the proposed surgery and previous non-compliance to medical procedures, concerns regarding the procedure and existence of reasons why one should not choose to undergo the surgery. The answers given by the patients allowed us to get acquainted with a variety of difficulties and concerns that arise as a result of their condition, thus acting a key role in the creation of the first questionnaire. This questionnaire maps the possible reasons for colostomy refusal. It was developed by the psychologist of our department. The refusal reasons were divided into four subgroups to observe what are the main subjects causing patients to decline the surgery. These subgroups included interpersonal and intrapersonal sensitivity, anxiety and depression and somatization. The second questionnaire is the Patient Health Questionnaire Mood Scale (PHQ-9) used to screen patients for depression in the non- psychiatric setting [16], and the third questionnaire is the Coping Behavior Questionnaire (COPE), designed to evaluate how the patients deal with stressful situations and negative events [17]. The second and third questionnaires are well known and validated questionnaires, used in various studies.

### Information process and statistical analysis

All statistical analysis was accomplished using IBM SPSS statistics program (Statistical Package for the Social Sciences) version 25.0, and Excel worksheet. The background characteristics are presented as means and standard deviation or as number of positive elements and percentage. A timeline for each patient disease course was designed using excel. The questionnaire process was accomplished with the psychologist of the surgical department.

## Results

Between 2019 and 2024, 96 patients were referred to our outpatient clinics with a diagnosis of rectal cancer. Out of them, 84 patients were treated with anterior resection with or without neoadjuvant treatment. One patient was treated with TAMIS for early rectal cancer. Eleven patients were advised to undergo an APR due to various reasons, mainly insufficient response to neo-adjuvant treatment that could not allow to proceed with a sphincter saving procedure.

Five of these patients completed an APR. However, six of them refused surgery and were included in our case series. Two patients out of the six died during the study period. Demographic data is displayed in Table 1.

**Table 1:** Background characteristics of the study population

Patient No	Age at diagnosis	Gender	Family status	TNM at diagnosis	Alternative treatments	Status
1	44	F	Married	T3N1M0	Yes	S/P APR
2	58	M	Married	T3N0M0	Yes	Follow up
3	46	M	Married/divorced	T3N0M0	Yes	Died
4	62	F	Single	T1N1M0	Yes	Follow up
5	57	M	Divorced	T3N0M0	No	Complete response
6	73	M	Married	T3N1M1	Yes	Died

**Fig. 1:**
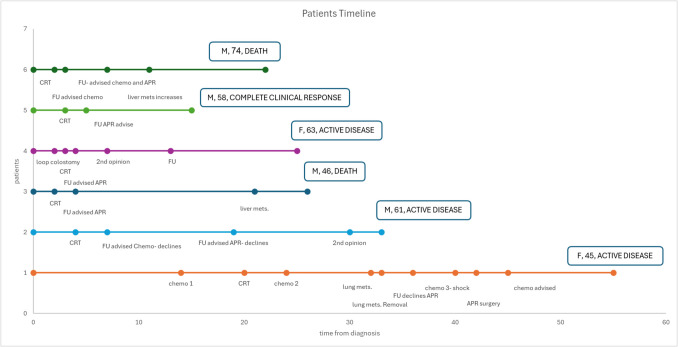
Long-term outcome and timeline events of patients refusing permanent colostomy

Figure 1 presents a diagram of the disease course for each patient. All patients started their treatment course with neoadjuvant treatment. After the initial therapy, and due to an incomplete response of the tumor, they were advised for a definitive surgery, namely APR. All of them refused an APR and continued inconsistent follow up.

Two patients (numbers 3 and 6) died during the study’s period after 26 and 22 months respectively. Patient 1 had two rounds of chemotherapy and one round of chemoradiation. She underwent a resection of single lung metastasis, and after an anaphylactic reaction in the third chemotherapy round, she relented and underwent an APR. Two other patients (number 2 and 4) dropped out of an orderly follow up after the chemoradiation, and after, they were advised for an APR. In light of their refusal for imaging tests, we do not know their oncological status. Patient 5 completed a chemoradiation course and had agreed for a follow up exam only during surgery for the repair of a symptomatic inguinal hernia. In the examination, the tumor has disappeared. Further imaging studies did not reveal an existing tumor and currently he is considered as a complete clinical responder.

### Interpretation of the three questioners

The results of the first questionnaire are displayed in Table 2. Four patients answered the first questionnaire, and five patients answered the second and third questionnaire. According to the first questionnaire, the two most common reasons for declining a colostomy were feelings of depression and anxiety related to the need to live with a stoma, a non-natural orifice, for the rest of their lives. Secondly, the fear and uneasiness related to inter-personal sensitivity (for example difficulty in social events). In the PHQ-9 questionnaire, one patient had moderate depression, two had mild and two had minimal depression. According to the COPE questionnaire, three patients were diagnosed as being more emotion focused when solving a problem, and two were diagnosed as being problem focused.

**Table 2:** Results of the questionnaire regarding possible reasons for colostomy refusal

Patient No	Inter-personal sensitivity (30 MAX)	Intra personal sensitivity (20 MAX)	Depression and anxiety (25 MAX)	Somatization (15 MAX)
1	29	16	23	10
2	11	11	24	9
4	15	14	21	7
6	28	16	21	11

## Discussion

During the years of the current study, 96 patients were diagnosed with rectal cancer. Eleven patients were advised for an APR (11.4%), and surprisingly more than 50% of them refused, knowing it would risk their lives. The aim of the current study was to shed a light on the characteristics of these patients, and to understand the reasons for declining a permanent colostomy, hopefully to provide a more tailored care in the future.

Rectal surgery refusal rates are low, and they change according to the stage of the disease, age, race, insurance status, and rural residency [13]. The exact rate of patients refusing specifically an APR with a permanent colostomy remains unknown. These patients often drop out of from follow-up and refuse to come for routine check-ups. The irregularity of routine check-ups and the transfer of patients between physicians make it challenging to determine their outcome.

The link between increased levels of depression and anxiety following colostomy has been previously documented [18]. Nevertheless, most of the ostomy patients dealing with anxiety or depression did not receive any psychological support [18]. The phenomena repeat itself in the current research—patients were diagnosed with different levels of depression. Depression and anxiety were found to be leading causes for stoma refusal. In addition, our patients were found to be more emotion focused when coping with a challenge. The emotional burden associated with the possibility of having a permanent stoma presented in the current and previous studies emphasizes the need for a psychological follow-up and psychological support for these patients.

Body image damage following rectal cancer surgery requiring a colostomy has been reported [19]. Many patients with permanent colostomy describe that they feel ashamed of their body, avoiding being seen in public, and feeling restricted [20]. In the current study, the primary reasons for refusing a colostomy were related to interpersonal sensitivity, highlighting the significant influence of public opinion on patients’ decisions. Patients described various difficulties, such as avoiding social gatherings, meeting new people, and exposing themselves in public places like the beach or at the pool. The patient who ultimately underwent the surgery, mentioned that she would have considered it more carefully if another patient in a similar situation had shared their firsthand experience. From this testimony, we concluded that pairing patients considering the surgery with those who have already undergone it might normalize the perception for prospective candidates. A support group could offer similar benefits and contribute to patients’ self-confidence. In addition, although we did not empirically test this during the current study, we believe in the importance of the relationship between the caregivers and the patients. We believe that a positive relationship between the caregivers and the patients would allow patients to maintain ongoing contact with their caregivers and therefore to be, to some extent, under follow up and potentially change their decision in the future. Further psychological research is needed to verify that statement, and perhaps even provide additional tools for therapists to provide the best response to patients facing the challenge described in our study.

The current study has several limitations. First, the sample size was small. This can be attributed to the fact that from the outset it was a very specific target population—only patients with low rectal tumors who were designated for an APR surgery and refused were included. Considering the advanced treatments for rectal cancer explained in the introduction, many patients who were previously designated for APR now have other treatment options. This allows for more tailored medicine and preservation of the rectum even in difficult conditions. Second, the interviews and questionnaires were conducted at a single point in time, after the completion of neoadjuvant treatment. There was no repetition of the questionnaires to see changes in the patients’ attitudes over time. Due to the patients’ reluctance to the health system, there was great difficulty in recruiting them even for one phase of the interviews, and certainly not for a second phase. However, we believe that it emphasizes the conclusions of our study, and the need for more empathy than usual when treating these patients. Lastly, one of the questionnaires used in the study was not a validated questionnaire, and was designed and interpreted by our research team and the psychologist of our department (M.A.).

## Conclusions

Undergoing an APR, which involves a permanent colostomy, is a challenging decision and experience, particularly for young patients. As caregivers, we should be mindful of these challenges when offering the surgery. Given that patients are often lost to follow-up, close monitoring should be considered even for those who firmly refuse the procedure. Additionally, psychological support should be provided throughout the entire process. Implementing tutoring programs, pairing patients who have had the surgery with those considering it, and forming support groups can offer valuable assistance and encouragement.

## Data Availability

No datasets were generated or analysed during the current study.

## References

[1] Benson AB, Venook AP, Al-Hawary MM et al (2022) Rectal cancer, Version 2.2022. NCCN Clin Pract Guide Oncol 20:1139 10.6004/jnccn.2022.0051 36240850

[2] Tilney HS, Heriot AG, Purkayastha S et al (2008) A national perspective on the decline of abdominoperineal resection for rectal cancer. Ann Surg 247:77 18156926 10.1097/SLA.0b013e31816076c3

[3] Conroy T, Bosset J-F, Etienne P-L et al (2021) Neoadjuvant chemotherapy with FOLFIRINOX and preoperative chemoradiotherapy for patients with locally advanced rectal cancer (UNICANCER-PRODIGE 23): a multicentre, randomised, open-label, phase 3 trial. Lancet Oncol 22:702 33862000 10.1016/S1470-2045(21)00079-6

[4] Atallah S, Albert M, Larach S (2010) Transanal minimally invasive surgery: a giant leap forward. Surg Endosc 24:2200 20174935 10.1007/s00464-010-0927-z

[5] Cercek A, Lumish M, Sinopoli J et al (2022) PD-1 blockade in mismatch repair–deficient, locally advanced rectal cancer. N Engl J Med 386:2363 35660797 10.1056/NEJMoa2201445 PMC9492301

[6] Rullier E, Laurent C, Bretagnol F et al (2005) Sphincter-saving resection for all rectal carcinomas. Ann Surg 241:465 15729069 10.1097/01.sla.0000154551.06768.e1 PMC1356985

[7] Garcia-Henriquez N, Galante DJ, Monson JRT (2020) Selection and outcomes in abdominoperineal resection. Front Oncol. 10.3389/fonc.2020.01339 33014775 10.3389/fonc.2020.01339 PMC7461900

[8] Stavropoulou A, Vlamakis D, Kaba E et al (2021) Living with a stoma: exploring the lived experience of patients with permanent colostomy. Int J Environ Res Public Health. 10.3390/ijerph18168512 34444262 10.3390/ijerph18168512 PMC8393572

[9] Vonk-Klaassen SM, De Vocht HM, Den Ouden MEM et al (2015) Ostomy-related problems and their impact on quality of life of colorectal cancer ostomates: a systematic review. 25. 10.1007/s11136-015-1050-3 10.1007/s11136-015-1050-3 PMC4706578 26123983

[10] Lee L, Trepanier M, Renaud J et al (2020) Patients’ preferences for sphincter preservation versus abdominoperineal resection for low rectal cancer. Surgery 169:623 32854970 10.1016/j.surg.2020.07.020

[11] Coffman AR, Tao R, Cohan JN et al (2021) Factors associated with the refusal of surgery and the associated impact on survival in patients with rectal cancer using the National Cancer Database. J Gastrointest Oncol 12:1482 34532104 10.21037/jgo-20-437 PMC8421873

[12] Hu X, Ye H, Yan W et al (2022) Factors associated with patient’s refusal of recommended cancer surgery: based on surveillance, epidemiology, and end results. Front Public Health. 10.3389/fpubh.2021.785602 35111717 10.3389/fpubh.2021.785602 PMC8801711

[13] Fields AC, Lu PW, Yoo J et al (2020) Treatment of stage I-III rectal cancer: who is refusing surgery? J Surg Oncol 121:990 32090341 10.1002/jso.25873

[14] Gordon HS, Street RL, Sharf BF et al (2006) Racial differences in doctors’ information-giving and patients’ participation. Cancer 107:1313 16909424 10.1002/cncr.22122

[15] Kennedy ED, Borowiec AM, Schmocker S et al (2018) Patient and physician preferences for nonoperative management for low rectal cancer: is it a reasonable treatment option? Dis Colon Rectum 61:1281 30239397 10.1097/DCR.0000000000001166

[16] Levis B, Benedetti A, Thombs BD et al (2019) Accuracy of Patient Health Questionnaire-9 (PHQ-9) for screening to detect major depression: individual participant data meta-analysis. BMJ 365:l1476 30967483 10.1136/bmj.l1476 PMC6454318

[17] Carver CS, Scheier MF, Weintraub JK (1989) Assessing coping strategies: a theoretically based approach. J Pers Soc Psychol 56:267–283 2926629 10.1037//0022-3514.56.2.267

[18] Ayaz-Alkaya S (2018) Overview of psychosocial problems in individuals with stoma: a review of literature. Int Wound J 16:243 30392194 10.1111/iwj.13018 PMC7948730

[19] Bullen TL, Sharpe L, Lawsin C et al (2012) Body image as a predictor of psychopathology in surgical patients with colorectal disease. J Psychosom Res 73:459 23148815 10.1016/j.jpsychores.2012.08.010

[20] González E, Holm K, Wennström B et al (2016) Self-reported wellbeing and body image after abdominoperineal excision for rectal cancer. Int J Colorectal Dis 31:1711 27506432 10.1007/s00384-016-2628-0 PMC5031731

